# Adoption of a Personal Health Record in the Digital Age: Cross-Sectional Study

**DOI:** 10.2196/22913

**Published:** 2020-10-28

**Authors:** Consuela Cheriece Yousef, Abin Thomas, Ahmed O Alenazi, Sumaya Elgadi, Laila Carolina Abu Esba, Aeshah AlAzmi, Abrar Fahad Alhameed, Ahmed Hattan, Saleh Almekhloof, Mohammed A AlShammary, Nazzal Abdullah Alanezi, Hani Solaiman Alhamdan, Manal Eldegeir, Rayf Abulezz, Sahal Khoshhal, Clara Glynis Masala, Omaima Ahmed

**Affiliations:** 1 Pharmaceutical Care Department Ministry of National Guard-Health Affairs Dammam Saudi Arabia; 2 King Saud bin Abdul Aziz University for Health Sciences Riyadh Saudi Arabia; 3 King Abdullah International Medical Research Center Riyadh Saudi Arabia; 4 Department of Biostatistics and Bioinformatics King Abdullah International Medical Research Center Riyadh Saudi Arabia; 5 Ministry of National Guard-Health Affairs Riyadh Saudi Arabia; 6 Department of Pharmacy Practice College of Pharmacy Princess Noura Bint Abdulrahman University Riyadh Saudi Arabia; 7 Pharmaceutical Care Department Ministry of National Guard-Health Affairs Riyadh Saudi Arabia; 8 Pharmaceutical Care Department Ministry of National Guard-Health Affairs Jeddah Saudi Arabia; 9 Pharmaceutical Care Department Ministry of National Guard-Health Affairs Madinah Saudi Arabia; 10 Pharmaceutical Care Department Ministry of National Guard-Health Affairs Al Ahsa Saudi Arabia; 11 Primary Health Care Prince Bader Housing Clinic Ministry of National Guard-Health Affairs Riyadh Saudi Arabia; 12 Qassim Primary Health Care Center Ministry of National Guard-Health Affairs Qassim Saudi Arabia; 13 Department of Pediatrics Ministry of National Guard-Health Affairs Dammam Saudi Arabia; 14 Department of Nursing Ministry of National Guard-Health Affairs Dammam Saudi Arabia; 15 Department of Pediatric Hematology/Oncology/BMT Ministry of National Guard-Health Affairs Jeddah Saudi Arabia

**Keywords:** patient portal, personal health record, eHealth, Middle East, Saudi Arabia

## Abstract

**Background:**

As health care organizations strive to improve health care access, quality, and costs, they have implemented patient-facing eHealth technologies such as personal health records to better engage patients in the management of their health. In the Kingdom of Saudi Arabia, eHealth is also growing in accordance with Vision 2030 and its National Transformation Program framework, creating a roadmap for increased quality and efficiency of the health care system and supporting the goal of patient-centered care.

**Objective:**

The aim of this study was to investigate the adoption of the personal health record of the Ministry of National Guard Health Affairs (MNGHA Care).

**Methods:**

A cross-sectional survey was conducted in adults visiting outpatient clinics in hospitals at the Ministry of National Guard Health Affairs hospitals in Riyadh, Jeddah, Dammam, Madinah, and Al Ahsa, and primary health care clinics in Riyadh and Qassim. The main outcome measure was self-reported use of MNGHA Care.

**Results:**

In the sample of 546 adult patients, 383 (70.1%) reported being users of MNGHA Care. MNGHA Care users were more likely to be younger (*P*<.001), high school or university educated (*P*<.001), employed (*P*<.001), have a chronic condition (*P*=.046), use the internet to search for health-related information (*P*<.001), and use health apps on their mobile phones (*P*<.001).

**Conclusions:**

The results of this study show that there is substantial interest for the use of MNGHA Care personal health record with 70% of participants self-reporting use. To confirm these findings, objective data from the portal usage logs are needed. Maximizing the potential of MNGHA Care supports patient engagement and is aligned with the national eHealth initiative to encourage the use of technology for high-quality, accessible patient-centered care. Future research should include health care provider perspectives, incorporate objective data, employ a mixed-methods approach, and use a theoretical framework.

## Introduction

There has been exponential growth in internet penetration globally, including in Saudi Arabia where the internet penetration rate is 93% and that of social media is 72% [1]. Living in the digital age, an increasing number of patients are empowered, computer-literate, and have access to the internet. With advances in health information technology and the now ubiquity of the internet, patients have access to health information and are expected to engage in their care in new ways. Consumer-based health apps have been developed to “transform the paternalistic model of health care into one that is responsive to consumer needs and treats each individual as a copilot in a life-long health care process” [2].

The eHealth movement, which is the delivery of health services and information through the internet or related technology, has been broadly promoted to improve the health status of patients [3,4]. Patient-facing tools have been endorsed by the Institute of Medicine and the World Health Organization to encourage patient- and person-centered care by facilitating patient involvement in medical decision-making [5,6]. The use of digital information for disease and health-related tracking is widespread and considered an “essential and important element in the health care sector” [7]. Innovative eHealth technologies such as personal health records (PHRs) are leveraged to support patients in becoming empowered. Health care organizations adopt PHRs to increase patient engagement in the drive to meet the triple aims of health care: increase access, reduce cost, and improve quality of care [8-10].

PHRs are either standalone, tethered, or integrated [11]. A standalone PHR is owned by the patient and allows a person to “access and coordinate their lifelong health information” [12]. A tethered PHR, or patient portal, is connected to an organization’s electronic health record (EHR) and allows patients to access information from their medical records [13]. An integrated PHR contains patient information from various sources such as pharmacy data, insurance claims, and an EHR. PHR features vary among health care organizations. Basic PHR features include viewing lab results, requesting prescription refills, and scheduling appointments [8,14,15]. More advanced PHR features include personal health-related reminders, secure messaging, eVisits, and social networking [16]. Since the terms “PHR” and “patient portal” have been used interchangeably in the literature, we here consider the two terms to be synonymous [9].

PHRs are designed to increase patient engagement in managing their health, increase care coordination, and to encourage patient empowerment [16-18]. Engaged patients monitor and update their medication and there is potentially more treatment concordance, leading to positive health outcomes [17,19]. Patient engagement is increasingly recognized as a vital component of safe, person-centered care [6]. Providing patients with access to their EHRs through a PHR is a method for health care systems to promote engagement [20].

Previous studies have shown that PHR use has positive effects on patient adherence, patient self-management skills, and clinical outcomes [16,19,21]. Wade-Vuturo et al [21] found that patients with diabetes mellitus who used a patient portal had better patient-provider communication, more satisfaction with care, greater self-management behaviors, and improved clinical outcomes (ie, hemoglobin A1c, hospital admissions, and emergency room visits). The PHR could bridge the gap due to the limited time and planning devoted to addressing chronic needs since acute issues are the focus of health care visits [22]. Despite the proposed benefits and consumer interest in PHRs, various studies have shown limited adoption and use [8,9,23-25]. According to Abd-Alrazaq et al [8], PHR adoption ranged from 0.13% in the United Kingdom to 10% in the United States.

PHRs are relatively new in the Middle East with less than 12% of health care organizations in the Arab world offering them [26]. Health care organizations in the Kingdom of Saudi Arabia (KSA) have only recently begun to implement PHRs. As with many developed countries, the KSA has invested substantial resources in the implementation of eHealth systems to reduce health care costs and improve care with 4 billion Saudi Arabian Riyals (SAR; US $1.1 billion) allocated by the government to improve eHealth [27]. The KSA has dedicated enormous funds to enhance national health care systems. To that end, the health care system is one of the priority areas of the National Transformation Program 2020 and Saudi Vision 2030, aiming to provide the highest quality of health care services to the citizens and residents while providing sufficient and efficient health care. The national eHealth initiative focused on improving the quality and efficiency of health care services by enhancing a patient-centered health care culture and increasing patient involvement in their care through technology [28]. In line with the KSA’s eHealth agenda, research on eHealth tools such as PHRs has grown [18,29-32].

The main objectives of this study were as follows: (1) to examine the prevalence of PHR use by region, (2) to categorize the PHR features used most frequently, and (3) to identify predictors of PHR adoption by patients. In addition, comparisons were made between portal users and nonusers according to (1) demographic and clinical characteristics, (2) access to and use of the internet, (3) health literacy and self-reported health status, and (4) online health-related information-seeking behavior.

Research into the actual use of the PHR focusing on the users and features accessed will lay the foundation for future developments of the system and targeted efforts to motivate patients to adopt its use. To reap the proposed benefits and maximize the return on investment, research must be conducted with an eye toward contextual factors and finding methods to promote initial and sustained PHR use among all patient populations.

## Methods

### Study Setting

The Ministry of National Guard Health Affairs (MNGHA) is a large tertiary health care system established in 1983 to provide state-of-the-art medical care to the National Guard’s soldiers and their dependents in all regions across the KSA [33]. The MNGHA is a leader in health care services in the Middle East. As a health care leader, MNGHA implemented its PHR known as MNGHA Care in 2018. Some of the features available in MNHGA Care include scheduling appointments, requesting medical reports, viewing radiology reports, checking laboratory results, requesting prescription refills, and providing vaccination reminders. MNHGA Care allows patients to upload personal health information such as weight, blood pressure, blood sugar, and exercise details. There is also a self-assessment feature where patients can enter information on pain control, performance status, and quality of life. In addition, it contains links to health educational information. MNGHA Care is a powerful tool that is expected to increase health awareness and promote positive health outcomes [34].

### Study Design

A cross-sectional survey design was used. An online survey was constructed and administered through QuestionPro. The study was conducted from December 2019 to February 2020.

### Sample

The target population consisted of adults who visited the outpatient waiting areas at Imam Abdulrahman Bin Faisal Hospital in Dammam, King Abdulaziz Medical City in Riyadh, King Abdulaziz Medical City in Jeddah, Prince Mohammad Bin AbdulAziz Hospital in Madinah, King Abdulaziz Hospital in Al Ahsa, and the primary health clinics in Riyadh and Qassim. The study was carried out at each site independently with each site’s research team. Patients or their caregivers were eligible to participate if they were aged 18 years and older and able to read and understand either Arabic or English.

### Sampling Strategy

Stratified random sampling with proportionate allocation was used to draw samples from the study population. The approximate number of patients seen daily in each city was used to determine the target sample for each site. Table 1 shows the proportionate allocation by city with the sample selected in proportion to the size of the population. Since no sampling frame was available, the biostatistician generated a random day and time schedule to be used by each site when beginning data collection.

**Table 1:** Proportionate allocation by region.

Region	Approximate number of patients/day	Minimum N
Dammam	250	27
Riyadh	1120	139
Jeddah	850	75
Medina	200	22
Al Ahsa	750	80

### Sample Size

The effect size was based on 40% internet usage for Usenet, listserv, discussion forums, internet phone, and streaming audio music [35]. This effect size was then applied to find the optimal sample size to detect a proportion of the Saudi population using the internet with a predefined accuracy. We assumed the minimum weekly visit frequency as the population to calculate the overall sample sizes. We further adjusted the sample size according to the proportion of daily visits in each region. With the above assumptions, the study required at least 364 complete records to estimate the proportion at a 95% confidence limit and within 5% precision.

### Participant Recruitment

Each research site used the survey time points and days from the table prepared prior to study initiation. A member of the research team recruited participants in the outpatient pharmacy waiting areas and used a password-protected device to allow the participants to access the survey through QuestionPro, provide consent, and complete the survey. All participants were informed about the study purpose, and were assured anonymity and confidentiality of the information collected. The research team member approached the subjects at randomly selected time points during active working hours until the minimum sample size requirement for the center was reached.

### Instrument

This study used a questionnaire developed by the lead author based on a literature review. The questionnaire contained 41 questions covering demographics, health status, satisfaction with health care, health literacy, mobile phone and internet usage, online health-related information-seeking behavior, and MNGHA Care. There were 13 statements rated on a Likert scale related to MNGHA Care use included but not analyzed for this study. The authors reviewed the questions to ensure readability and appropriateness. The questionnaire was forward-translated by native Arabic speakers and was back-translated by a professional translator and compared to the original. Before study initiation, a pilot test was performed in 20 volunteers at Imam Abdulrahman Bin Faisal Hospital with slight modifications made.

### Measures

Demographic and clinical characteristics were collected to describe the study sample and were self-reported. Demographic characteristics included health care facility, age, gender, marital status, educational level, employment status, and monthly household income. Clinical characteristics included the presence of a medical condition, number and type of medical conditions, self-reported health status, recent hospitalization (<6 months), recent emergency department visit (<6 months), and satisfaction with health care.

To characterize mobile phone and internet usage, one question was asked about smartphone ownership and another question was asked about the frequency of internet use.

Previous researchers have identified a link between health literacy and technology use [36,37].

Therefore, a single-item health literacy screener was used with the following question: “How often do you need to have someone help you when you read instructions, pamphlets, or other written material from your doctor or pharmacy?” [38].

Four questions were related to internet use for seeking health information: (1) Do you use the internet to search for medical information? (2) Are you a member of an online health community? (3) Do you discuss health issues on social media (eg, Facebook, Twitter)? (4) Do you use health apps on your mobile phone?

### Outcome

The outcome variable was the patient-reported use of the PHR, operationalized by asking the patients whether or not they used MNGHA Care.

### Ethics Statement

All participants were informed about the aim of the study and their right to not answer any question they felt uncomfortable answering. The study was approved by the Institutional Review Board (RD19/002/D) at King Abdullah International Medical Research Center in Riyadh, Saudi Arabia.

### Data Analysis

The raw data were downloaded from QuestionPro into Microsoft Excel and were analyzed using SAS 9.4. The proportion of PHR users with the corresponding 95% confidence limit was calculated using the Wilson score method. Descriptive analysis was used to summarize the categorical variables as frequency and percentage and continuous variables as mean and standard deviation. For reporting convenience, the continuous variables were grouped and the frequency and percentage are reported in each group. Further, we compared the relative frequency distribution of all variables across PHR users and nonusers. The chi-square test was used to measure the significance of the association between individual covariates and PHR usage. Finally, multiple logistic regression was used to estimate the odds ratio and the corresponding 95% confidence limit. Throughout the study, we considered any *P* value less than .05 as evidence for a significant result.

## Results

### Sample Characteristics

The baseline characteristics of the participants are shown in Table 2. A total of 546 participants agreed to complete the survey. Most of the participants were men, married, employed, and university graduates. The mean age was 37.39 (SD 11.23) years. The estimated monthly income was greater than 5000 SAR/month (US $1333/month) for 68.0% (335/493) of the participants. Most participants rated their health as very good or excellent. The majority reported only one medical condition. The most frequently reported medical conditions were diabetes, hypertension, and asthma or chronic obstructive pulmonary disease. About a quarter of the sample reported being hospitalized and approximately half had visited the emergency department in the previous 6 months. All patients were either satisfied or very satisfied with their health care. Most patients either never or rarely need help with reading materials from their physician or pharmacist.

**Table 2:** Baseline demographics of participants (N=546).

Variable		n (%)
**Age (years)**		
	18-29	133 (26.3)
	30-39	170 (33.6)
	40-50	104 (20.6)
	>50	99 (19.57)
Male		280 (53.3)
**Health care facility**		
	King Abdulaziz Medical City (Riyadh)	118 (21.6)
	King Abdullah Specialized Children’s Hospital (Riyadh)	35 (6.4)
	Primary Health Clinic (Riyadh)	32 (5.9)
	King Khaled National Guard Hospital (Jeddah)	124 (22.7)
	Prince Mohammed Bin Abdulaziz Hospital (Madinah)	32 (5.9)
	King Abdulaziz Hospital (Al Ahsa)	145 (26.6)
	Imam Abdulrahman Bin Faisal Hospital (Dammam)	27 (4.9)
	Primary Health Clinic (Qassim)	33 (6.0)
Married		426 (78.9)
**Education**		
	Elementary or less	65 (11.9)
	Middle school	44 (8.1)
	High school	177 (32.6)
	University	233 (41.1)
	Postgraduate	34 (6.3)
Employed		273 (45.3)
**Health status**		
	Excellent	224 (41.0)
	Very good	188 (34.3)
	Good	94 (17.2)
	Fair	27 (5.0)
	Poor	13 (2.4)
**Number of medical conditions**		
	0	152 (27.9)
	1	280 (51.5)
	≥2	112 (20.6)
**Type of medical condition**		
	Diabetes	118 (21.6)
	Hypertension	104 (19.0)
	Asthma or COPD^a^	60 (11.0)
**Satisfaction with health care**		
	Very satisfied	502 (92.5)
	Satisfied	41 (7.6)
Hospitalized within the last 6 months		133 (24.4)
Visited the emergency department within the last 6 months		249 (45.9)
**Need help with reading instructions**		
	Never	334 (61.2)
	Rarely	110 (20.1)
	Sometimes	81 (14.9)
	Often	16 (2.9)
	Always	4 (0.7)

^a^COPD: chronic obstructive pulmonary disease.

### Internet Use and Online Health Information–Seeking Behavior

Table 3 shows data from the survey related to internet use and online health information–seeking behavior. An overwhelming majority of the participants reported using a smartphone and accessing the internet several times a day. A large majority also reported using the internet to search for medical information. However, relatively few participants reported being a member of an online health community or discussing health issues on social media. Over half the participants use health apps on their mobile phones.

**Table 3:** Internet use and online health information–seeking behavior (N=546).

Internet use category		n (%)
Smartphone use		516 (94.5)
**Frequency of internet use**		
	Several times a day	442 (81.0)
	About once daily	30 (5.5)
	A few times per week	19 (3.5)
	A few times per month	22 (4.0)
	Rarely or not at all	29 (5.3)
Use the internet to search for medical information		381 (69.8)
Member of online health community		44 (8.1)
Discuss health issues on social media		101 (18.5)
Use health apps on your mobile phone		299 (54.8)

### MNGHA Care Use

**Figure 1:**
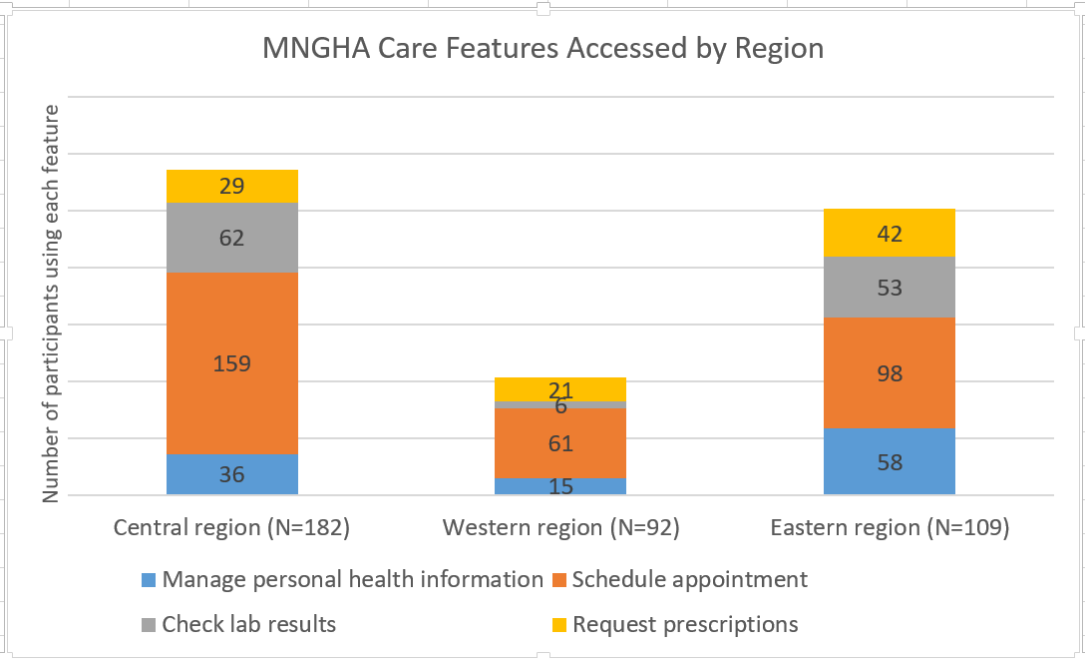
MNGHA Care features accessed by region.

Of the 546 participants, 460 (84.7%) were aware of MNGHA Care. As shown in Table 4, most participants were made aware of MNGHA Care through someone from the organization—health care provider or other hospital staff—followed by a family member. Of the 460 participants aware of the PHR, 383 (83.3%) reported using it. The health care facilities were classified into the central (Riyadh and Qassim), eastern (Al Ahsa and Dammam), and western (Jeddah and Madinah) regions. There was a statistically significant difference in use by region (*P*<.001): central region (182/383, 47.5%), eastern region (109/383, 28.5%), and western region (92/383, 24.0%). In response to the question “How long have you been using MNGHA Care?”, the majority of respondents (267/383, 69.7%) reported using it for the past 12 months with most (160/383, 41.8%) using it only within the last 6 months. Most participants reported using MNGHA Care a few times a month (210/383, 54.8%) to rarely (119/383, 31.1%). PHR features accessed by the participants are shown in Figure 1. Scheduling appointments was the feature used most frequently in all regions. In the western region, use of the PHR to check laboratory results was quite low (6.5%) compared to that reported in the eastern region (48.6%) and central region (34.1%). Prescription refill requests were used the most in the eastern region (38.5%), followed by the western region (22.8%) and central region (15.9%).

**Table 4:** Source of MNGHA Care recommendation among users (N=383).

Recommender	n (%)
Health care provider	179 (46.7)
Other hospital staff	41 (10.7)
Family	90 (23.5)
Friends	47 (12.3)
No one	20 (5.2)

Table 5 shows a comparison of the baseline demographics between MNGHA Care users and nonusers. There were statistically significant differences between the groups with respect to age, educational level, and employment status. MNGHA Care users were younger (18-39 years), had a high school or university education, had at least one medical condition, and larger monthly household incomes relative to those of nonusers.

**Table 5:** Demographic characteristics of personal health record (PHR) users and nonusers.

Characteristic		PHR user, n (%)	PHR nonuser, n (%)	*P* value
**Age group (N=487)**				.001
	18-29 years	102 (33.0)	30 (21.3)	
	30-39 years	128 (41.4)	42 (29.8)	
	40-49 years	79 (22.8)	25 (17.7)	
	≥50 years	37 (10.7)	44 (31.2)	
**Gender (N=523)**				.44
	Male	194 (52.4)	88 (56.2)	
	Female	176 (47.6)	67 (43.8)	
**Marital status (N=537)**				.30
	Married	303 (80.2)	121 (76.1)	
	Unmarried	75 (19.8)	38 (23.9)	
**Educational level (N=540)**				<.001
	Elementary school or less	26 (6.8)	38 (23.9)	
	Middle school	25 (6.6)	18 (11.3)	
	High school	139 (36.5)	38 (23.9)	
	University	167 (43.8)	55 (34.6)	
	Postgraduate	24 (6.3)	10 (6.3)	
**Monthly household income (N=490)**				.11
	<1333 US $/month	105 (30.1)	54 (37.6)	
	1333-2665 US $/month	118 (33.8)	50 (35.5)	
	>2666 US $/month	127 (36.1)	38 (27.0)	
**Employment status (N=536)**				<.001
	Employed	207 (54.6)	59 (37.6)	
	Unemployed	115 (30.3)	55 (35.0)	
	Retired	36 (9.5)	34 (21.7)	
	Student	21 (5.5)	9 (5.7)	

Table 6 compares MNGHA Care users and nonusers according to health status. Importantly, the frequency of users among those with a medical condition was more than double that of users without a medical condition, representing a significant difference between users and nonusers according to medical condition.

**Table 6:** Health status of MNGHA Care users and nonusers.

Question		Users		Nonusers		*P* value	
**How do you rate your health? (N=543)**						<.001	
	Poor		5 (1.3)		8 (5.0)		
	Fair		13 (3.4)		14 (8.8)		
	Good		54 (14.1)		39 (24.4)		
	Excellent		170 (44.4)		53 (33.1)		
**Do you have any medical condition? (N=541)**						.046	
	Yes		265 (69.4)		124 (78.0)		
	No		117 (30.6)		35 (22.0)		
**Number of medical conditions (N=541)**						.002	
	0		117 (30.6)		35 (22.0)		
	1		201 (52.6)		77 (48.4)		
	≥2		64 (16.8)		47 (29.6)		
**Hospitalized within the last 6 months (N=542)**						.04	
	Yes		84 (21.9)		49 (30.8)		
	No		299 (78.1)		110 (69.2)		
**Visited the emergency department within the last 6 months (N=545)**						.51	
	Yes		179 (47.1)		70 (43.8)		
	No		201 (52.9)		90 (56.3)		

Table 7 compares MNGHA Care users and nonusers according to health literacy and online health-related information-seeking behavior. There were statistically significant differences in PHR use in those who use the internet to search for medical information (*P*<.001) and in those who use health apps on their mobile phone (*P*<.001).

**Table 7:** Health literacy and online health information-seeking behavior.

Question		PHR^a^ users, n (%)	PHR nonusers, n (%)	*P* value
**How often do you need to have someone help you when you read instructions, pamphlets, or other written material from your doctor or pharmacy? (N=539)**				.47
	Never	230 (60.1)	102 (64.2)	
	Rarely	85 (22.2)	25 (15.7)	
	Sometimes	53 (13.8)	27 (17.0)	
	Often	12 (3.1)	4 (2.5)	
	Always	3 (0.8)	1 (0.6)	
**Do you use the internet to search for medical information? (N=543)**				<.001
	Yes	303 (79.1)	77 (48.1)	
	No	80 (20.9)	83 (51.9)	
**Are you a member of an online health community? (N=542)**				.61
	Yes	33 (8.6)	11 (6.9)	
	No	350 (91.4)	148 (93.1)	
**Do you discuss health issues on social media (e.g., Facebook, Twitter) (N=541)**				.07
	Yes	79 (20.7)	22 (13.8)	
	No	302 (79.3)	138 (86.3)	
**Do you use health apps on your mobile phone? (N=533)**				<.001
	Yes	250 (65.6)	48 (31.6)	
	No	131 (34.4)	104 (68.4)	

^a^PHR: personal health record.

Table 8 shows the logistic regression results assessing the influence of various participant characteristics on MNGHA Care use. Of the 9 predictor variables, only 3 were statistically significant: educational level, use of the internet to search for health-related information, and use of health apps on the mobile phone. Although higher educational level was associated with more frequent PHR use, this relationship was only statistically significant for patients with a high school education, who had a 4.08-times higher odds of using the PHR compared with those having an elementary education (*P*=.002). Patients who use the internet to search for health-related information had a 2.4-times higher odds of using the PHR (*P*=.005). Finally, patients who use health apps on their mobile phones had a 2.1-times higher odds of using the PHR (*P*=.008).

**Table 8:** Predictors of MNGHA Care use.

Variable		Odds ratio (95% CI)		*P* value	
Female		1.426 (0.739-2.750)			.29
**Age (years)**					
	18-29		Reference		
	30-39		0.738 (0.335-1.628)		.59
	40-49		0.657 (0.258-1.672)		.99
	≥50		0.378 (0.129-1.105)		.08
**Employment status**					
	Unemployed		Reference		
	Employed		1.083 (0.457-2.569)		.39
	Retired		0.703 (0.241-2.051)		.65
	Student		0.640 (0.182-2.250)		.58
**Household income (US $/month)**					
	<1333		Reference		
	1333-2665		1.044 (0.491-2.222)		.53
	>2666		1.571 (0.641-3.849)		.23
**Region**					
	Central		Reference		
	Eastern		0.199 (0.098-0.406)		.09
	Northern		0.521 (0.088-3.081)		.49
	Southern		0.148 (0.038-0.567)		.14
	Western		0.225 (0.105-0.480)		.22
**Education**					
	Elementary school or less		Reference		
	Middle school		2.29 (0.695-7.549)		.58
	High school		4.08 (1.428-11.662)		.002
	University		1.825 (0.590-5.646)		.99
	Postgraduate		1.170 (0.267-5.138)		.33
**Number of medical conditions**					
	0		Reference		
	1		1.323 (0.722-2.425)		.95
	≥2		1.695 (0.740-3.88)		.28
Internet use for health-related information			2.448 (1.32-4.539)		.005
Health apps on mobile phones			2.069 (1.209-3.539)		.008

## Discussion

### Principal Findings

In the sample of 546 adult patients, 383 (70.1%) reported being users of MNGHA Care. The central region (Riyadh and Qassim) had higher use (83.4%) than the western (Jeddah and Madinah, 59.4%) or eastern (Dammam and Al Ahsa, 64.1%) regions. There were 460 participants (84.6%) who were aware of the PHR. Of those, the majority (83.3%) reported using MNHGHA Care. Despite the high penetration of internet (93%) and social media (72%) use in the KSA, few participants reported being a member of an online health community (8.0%) or discussing health issues on social media (18.6%), whereas a high proportion of participants reported using the internet to seek health information [1]. Other researchers in the KSA have found a growing interest in patients using online social networking for health-seeking purposes [39-41].

Prior to the implementation of MNGHA Care in 2018, Al-Sahan and Saddik [31] conducted a study among 424 patients in the outpatient setting at MNGHA Riyadh to gauge the acceptance of the PHR. In their study, most patients were interested (25.2%) or very interested (60.6%) in a PHR. The results of this study appear to be concordant with these previous findings, even though their sample was predominantly female (68.2%) with the majority having no medical condition (68.2%). Technology use was also evaluated, demonstrating high internet use (95.9%), smart device use (92.2%), and computer use (80.7%), with only 15.9% accessing patient electronic services from the MNGHA website (15.9%). Our study showed similar findings with 94.5% having a smartphone and 81% using the internet several times a day.

MNGHA Care users were more likely to be younger (18-39 years of age), high school or university educated, employed, users of the internet to search for health-related information, and users of health apps on their mobile phones. Most PHR studies have been conducted in Western countries and have noted differences in PHR use by age, gender, and ethnic background, with people of a lower socioeconomic status using PHRs less often [19,42-47]. In the systematic review conducted by Abd-Alrazaq et al [8], the factors positively associated with use of a PHR were awareness of the PHR, perceived ease of use, perceived usefulness, internet access, income, and education level. Our study also showed a positive association of PHR use with awareness, internet access, income, and educational level.

Our results indicated that increasing age is associated with lower odds of using the PHR. This is consistent with the existing literature [44,46,48-51]. Even though older patients have more chronic conditions and are in the greatest need of support in disease self-management, many do not use a PHR for a variety of reasons. Disparities in PHR use by age have frequently been cited in the literature with many studies showing that older individuals are less likely to use a PHR. Confounding factors include low computer literacy, low eHealth literacy, or less inclination to use technology, ultimately leading to more difficulties with advanced technologies such as PHRs [17,32,48]. In a study using a simulated PHR in adults aged 40 years and above, the authors concluded that adults with age-related declines in reasoning and cognitive abilities were more likely to have difficulties completing more complex health management tasks using a PHR [52]. In a study conducted in Saudi Arabia using a simulated PHR to perform various health management tasks, the authors found an increased probability to watch the help video with each 1-year increase in age, but did not note difficulties by age in completing simple compared to complex tasks [32]. The authors suggested embedding aids such as help videos in PHRs to improve the comprehension of numeric health information [53]. Special considerations should be made in the design of a PHR with targeted training sessions for the older population. Allowing patients to participate in the design of the PHR through focus groups is a strategy employed by some health care organizations [50].

Another area where the digital divide has been evident with the use of PHRs relates to education level [9,42,54]. Our study showed that a high school or university education was associated with use of the PHR; however, there was no difference in use for those with a postgraduate degree. This is inconsistent with previous research, which has shown increasing PHR use with higher levels of education. In the systematic review conducted by Zhao et al [9] examining barriers and facilitators to PHR use, the authors recognized that one of the most common barriers is lack of a user-friendly interface. They identified 17 studies that mentioned redesigning the PHR and patient portal interfaces so that they are “easy-to use, easy-to-navigate interfaces and simpler language” [9]. Honein-AbouHaidar et al [26] evaluated the acceptance of the patient portal in Lebanon and noted the importance of simplifying and tailoring messages to the target population. Focusing on the patients and how the information is presented in the PHR will prevent the widening of health disparities.

Other studies have found that patients with chronic medical conditions are more frequent PHR users than those without [26,44,51,55]. In this study, 389 (71.9%) of the participants had a medical condition, 265 (68.1%) of whom reported using MNGHA Care. Similar to the literature, patients with a chronic medical condition had a higher prevalence of PHR use. Diabetes mellitus was the most frequently reported medical condition in our participants. Numerous studies have evaluated PHR use in this population to improve diabetes care, increase self-management, and optimize health outcomes [14,21,37,49,56-61]. Belcher et al [29] conducted a 12-week study in 31 patients with diabetes mellitus in the eastern province of Saudi Arabia who were sent twice-weekly messages through the patient portal. They found a reduction in hemoglobin A1c (11% to 9%) and fasting blood sugar (198 to 173 mg/dL). Secure messaging, a feature not available in MNGHA Care, has been associated with improved glycemic control in studies of PHR use in patients with diabetes mellitus [13]. In the study by Al Sahan and Sadek [31], 74.1% of participants reported a desire to communicate with a physician. Since patients showed an interest in secure messaging and studies have shown positive patient outcomes, it may be the right time to consider adding this feature in phases across the organization to support patient-centered communication.

This study showed that the MNGHA Care feature used most commonly was appointment scheduling (83.0%). In the study by Al Sahan and Sadek [31], participants were interested in accessing laboratory results (91.7%), radiology results (82.9%), and appointment scheduling (90.5%). With real-world use, our participants underutilized the features for managing personal health information (28.5%), checking laboratory results (31.6%), and requesting prescription refills (24.0%). Although the previous study showed a high level of patient interest in specific PHR features 2 years before MNGHA Care was implemented in the organization, utilization was less than would be expected after the implementation. Indeed, interest in PHRs and their features has been found to be higher than the actual adoption, with 80% of US respondents surveyed indicating interest in PHRs but only 2.2% of the population actually used a PHR [53]. Having the technology available is one challenge to be overcome but it is also necessary to monitor the process, find ways to connect patients to the technology, and investigate their concerns regularly postimplementation.

An explanation for the low percentage of participants requesting prescription refills is that the pharmacies in each region chose when to activate this feature. Not all pharmacies in all regions had activated the refill function at the time of performing this study. With respect to checking laboratory results and managing personal information, patients may have disliked the interface or had problems interacting with the system. Zhao et al [9] highlighted the need for health care providers to work with patients and demonstrate the features as a way to increase the value of the PHR from the patients’ perspective. There also could have been differences in the way the PHR was rolled out in each region. The central region possibly implemented more strategies aimed at encouraging patient awareness compared to the other regions. Finally, it is recognized that health care providers play an influential role in endorsing PHR use [8,9,44,47]. This is evident in our study, in which the health care provider (47.9%) or hospital staff (10.8%) was responsible for recommending the use of MNGHA Care to most participants. For better utility of the PHR, all health care providers and staff need proper training to support their patients. It is hoped that an impact of this study will be to disseminate information on the availability and benefits of the PHR and its various functions to patients and health care providers.

### Limitations

A major limitation of this study is the use of only self-reported data. Several biases are associated with self-reported data, including social desirability, recall, and nonresponse bias [62]. There may have been overreporting of PHR use by participants because they felt that this was the answer expected of them. In the systematic literature review and meta-analysis conducted by Fraccaro et al [25], the researchers found an adoption rate of 71% in controlled experiments and 23% in real-world experiments. In a cross-sectional study conducted in the Netherlands, there was a 32.1% adoption rate [44]. Objective PHR data from system usage logs can be used along with self-reported data; however, such data were not available to the research team for this study. Systems data would provide information on the total MNGHA population registered to use the PHR, patient logins, and use of specific features. In addition, those who agreed to participate may have been more likely to use MNGHA Care compared with those who did not participate, resulting in skewed results. Another limitation is the use of only a quantitative approach for analysis. This study would have benefited from combining quantitative data with qualitative data in a mixed-methods approach. The advantage would be gaining greater insight into patient acceptance and concerns with use of MNGHA Care. Such analysis would allow for a more in-depth examination of some of the barriers and facilitators to adoption within the KSA. Another limitation is that the study was conducted in a single health system. However, the large sample size of patients from across the country who were visiting different departments should increase the generalizability of the findings. Finally, the lack of a theoretical framework possibly limits the interpretation of interrelationships between patient factors and PHR characteristics.

### Conclusions

The results of this study show that there is a great deal of interest in use of MNGHA Care with 70% of participants self-reporting use. To our knowledge, this study is the first to report on the adoption of a PHR in a real-world setting in the KSA. To confirm these findings, objective data from the portal usage logs are needed. With the COVID-19 pandemic, the entire world is learning many lessons as the eHealth landscape transforms rapidly. One lesson we are learning is how to engage in health care remotely and efficiently via electronic apps. Remote personal health engagement has become the new normal. Maximizing the potential of MNGHA Care supports patient engagement and is aligned with the national eHealth initiative to encourage the use of technology for high-quality, accessible patient-centered care. Future research should include health care provider perspectives, incorporate objective data, employ a mixed-methods approach, and use a theoretical framework.

## Abbreviations

EHR: electronic health record
KSA: Kingdom of Saudi Arabia
MNGHA: Ministry of National Guard Health Affairs
PHR: personal health record
SAR: Saudi Arabian Riyal
